# A Toxic Alkaloid Constantly Exhaled from the Lungs

**Published:** 1888-04

**Authors:** 


### *A TOXIC ALKALOID CONSTANTLY EXHALED FROM THE LUNGS.*

Perhaps the most important medical discovery made during the past year is that of Drs. Brown-Sequard and D'Arsonval, who have demonstrated that a toxic substance, probably an alkaloid, is constantly exhaled from the lungs of mammals. These scientists injected under the skin, in seven rabbits, from twenty to forty-four centigrammes of water obtained by condensing the vapor exhaled from the lungs of dogs. Four of the rabbits died within twenty-four hours, one in thirty-six hours, and the remaining two were still living, but very feeble, when the communication was made to the Academy of Sciences, January 16th. The symptoms generally produced were slowing of the respiration, increased rapidity of the cardiac pulsations, general paresis, and diarrhœa. *Post mortem* examination shows ecchymoses in the lungs, and the intestine filled with a yellowish fluid. After showing that this condensed pulmonary vapor had a toxic effect, the experimenters next endeavor to ascertain the nature of the poison, whether it is chemical or microbic. This was determined by boiling the condensed pulmonary vapor, under pressure, in closed flasks. In this way, all micro-organisms were destroyed, and the fluid thus sterilized was injected into rabbits without any diminution of the poisonous properties. Brown-Sequard even thinks that the toxicity was increased. They conclude, from the following facts, that the expired poison is an alkaloid resembling the ptomaines and leucomaines :

1. The pulmonary fluid containing the poison is alkaline.

2. The toxicity is not destroyed by heat or boiling in a closed flask.

3. From the symptoms produced when the substance is injected into the blood or tissue of rabbits.

The demonstration of the existence of this poison is of the greatest importance, and the discoverers promise to make some experiments showing the effects of the continued inhalation of small quantities, thus throwing new light upon the etiology of diseases thought to be induced by inadequate ventilation.

---

In concluding his annual address before the Philadelphia County Medical Society, January, 1888, the president, Dr. J. Solis-Cohen, commented as follows on the relations of the profession to the public : " It is to be deplored, on the score of professional ethics, that mention of some of our scientific work is occasionally noted unofficially in the public newspapers, despite the express interdiction in our by-laws. Whether this prohibitory clause be deemed judicious or not, it is plainly the duty of all members to accede to its behests. Those who disapprove of it should present their reasons for doing so in full meeting, and should endeavor to have it rescinded. They have neither the right to ignore it, on the one hand, nor the right to disobey it, on the other. On several occasions, when reporters of the daily press have been present at our meetings, they have assured me that they were present on invitation of a member; that they had no desire to intrude, and had believed that their presence would be agreeable to the Society. While there is no reason to doubt that much of the individual editorial advertisement of subjects discussed, or to be discussed at societies, or of operations performed, or to be performed, in public places or in private, is due to officiousness on the part of a student, a follower, an attendant, or a patient, there is equally good reason to believe that most of it is courted, directly or indirectly, by the individual most interested. Of this fact I have been amply assured by newspaper men, who have been my own patients, and to whom I have put the question direct. I have been assured, further, that it could be taken for granted that little matter of personal medical importance ever gains access to the papers without the knowledge of those most intimately concerned.

---

Dr. Frank H. Potter, Lecturer on Laryngology in the Medical Department of Niagara University, has removed his office and residence to 273 Franklin street. This is a more central location than the doctor's former place, and the house is well adapted to carrying on of his especial work.

Dr. Edward Clark, formerly of Huron street, has also removed to 271 Franklin street.

Dr. Charles G. Stockton, Professor of Theory and Practice of Medicine in the Buffalo Medical College, has removed to 278 Franklin street. There are now eight doctors on this one block, and yet it is reported the neighborhood is healthy.

THURSDAY, March 24, 1888, was a cheerful occasion for many friends of the *Western Pennsylvania Medical College*, in Pittsburgh. The Grand Opera House was crowded with those on hand to extend congratulations, and to aid in commemoration of the second annual commencement. Thirty-four graduates received the degree of M. D., being an increase of exactly fifty per cent. above last year's number.

---

WE take pleasure in calling the attention of our readers to the advertisement of Messrs. John Wyeth & Bro., on page 4. The new remedies they bring forward in the form of their compressed tablets will be of interest to every physician. The circular matter they offer to supply is very concise, and collated with much acumen, and shows an evident thorough knowledge of the therapeutic value of these recent valuable antipyretics and antiseptics.

